# The Role of the Broader Autism Phenotype and Environmental Stressors in the Adjustment of Siblings of Children with Autism Spectrum Disorders in Taiwan and the United Kingdom

**DOI:** 10.1007/s10803-017-3134-0

**Published:** 2017-05-13

**Authors:** Hsiao-Wei Joy Tsai, Katie Cebula, Sue Fletcher-Watson

**Affiliations:** 1 Moray House School of Education, St John’s Land, The University of Edinburgh, Holyrood Rd, Edinburgh, EH8 8AQ UK; 2 The Patrick Wild Centre, Centre for Clinical Brain Sciences, The University of Edinburgh, Kennedy Tower, Edinburgh, EH10 5HF UK

**Keywords:** Autism, Broader autism phenotype, Typically developing sibling, Adjustment, Cross-culture

## Abstract

The influence of the broader autism phenotype (BAP) on the adjustment of siblings of children with autism has previously been researched mainly in Western cultures. The present research evaluated a diathesis-stress model of sibling adjustment using a questionnaire study including 80 and 75 mother-typically developing sibling dyads in Taiwan and the United Kingdom (UK). UK siblings reported elevated adjustment difficulties compared to the Taiwanese sample and to normative data. Whilst higher BAP levels were generally associated with greater adjustment difficulties, differences were found across cultures and respondents. Although significant diathesis-stress interactions were found, these were in the opposite direction from those predicted by the model, and differed across cultural settings. Implications for culturally-sensitive sibling support are considered.

## Introduction

Siblings of children with autism spectrum disorders (ASD) have often been found to be more susceptible to psychological maladjustment than siblings of typically developing children (Lovell and Wetherell 2016; Petalas et al. 2012; Walton and Ingersoll 2015; Griffith et al. 2014). In contrast, some research has found that typically developing sibling of children with autism (TD sibling) displayed better social competence or positive self-concept (Verte et al. 2003; Macks and Reeve 2007; Kaminsky and Dewey 2002) and others have found no difference in comparison to siblings of TD children or normative data (Tomeny et al. 2012; Quintero and McIntyre 2010; Rodgers et al. 2016; Dempsey et al. 2012).

Several demographic variables have been identified as having an association with TD siblings’ adjustment, such as socioeconomic status, family size, gender and age (Kaminsky and Dewey 2002; Verte et al. 2003; Macks and Reeve 2007; Giallo and Gavidia-Payne 2006). Psychological variables that may moderate or mediate adjustment difficulties of siblings of children with ASD have also been studied, such as social support, impact of life events and coping strategies (Petalas et al. 2012; Hastings 2003; Ross and Cuskelly 2006). In particular, the severity of symptoms of the child with ASD and their challenging behaviour have consistently been found to be a predictor of TD siblings’ psychological well-being (Benson and Karlof 2008; Lyons et al. 2010; Meyer et al. 2011) and this may go some way to explaining the variability in results of studies examining sibling outcomes.

Over recent years ASD family research has moved away from looking simply at whether siblings experience positive or negative outcomes, towards a more theoretical driven consideration of the pathways to such outcome (e.g. McHale et al. 2016). In particular, there has been a more integrated examination of genetic vulnerabilities and how they interact with environmental stressors to influence TD sibling adjustment (Petalas et al. 2012; Mohammadi and Zarafshan 2014; Meyer et al. 2011; Walton and Ingersoll 2015). The principal genetic vulnerability factor of interest in the present study is the broader autism phenotype (BAP). The BAP is a collection of behaviours and traits that are conceptually similar to the core ASD symptom domains, but are a sub-clinical manifestation of such traits (Folstein and Rutter 1977; Piven et al. 1997; Cruz et al. 2013). It has been estimated that between 12 and 20% of the non-autistic siblings of children with ASD display such traits (Rotatori and Deisinger 2015).

Multiple studies have found that siblings of children with ASD are more likely to have subtle difficulties in communication (Ben-Yizhak et al. 2011; Gamliel et al. 2009), social interaction, and academic development (Constantino et al. 2006; Yoder et al. 2009) or to exhibit neurocognitive impairments (Dawson et al. 2002) compared to siblings of TD children. However, environmental influences will also play a role. For example, Barak-Levy et al. (2010) found that TD siblings of children with ASD participated less in extracurricular activities, and had poorer social relations, when compared to siblings of TD children. As suggested by the authors, whilst genetically-based traits may lead TD siblings to be more introverted and less active than other children, the presence of a child with ASD at home might also make it more difficult to develop social relations.

Bauminger and Yirmiya (2001) proposed using a ‘diathesis-stress’ model for research with siblings of children with ASD. This model incorporates the influence of the genetic vulnerability (diathesis) and its interaction with environmental stress to impact on families of individuals with ASD. This model has been adopted in several sibling studies to date (Orsmond and Seltzer 2009; Petalas et al. 2012; Walton and Ingersoll 2015; Mohammadi and Zarafshan 2014). These have provided partial support for the diathesis-stress model, finding that environmental stressors (e.g. the presence of stressful life events, or symptom severity in the child with ASD) interacts with TD sibling BAP level to influence outcomes such as their emotional symptoms, adjustment outcome and sibling relationship. Such findings can inform support practices for the siblings of children with autism by highlighting relevant sibling traits which should be taken into account when providing support, as well as by identifying direct targets for intervention.

However, while research has been gradually begun to identify the factors, such as BAP, that contribute to TD sibling adjustment, the majority of research to date has been based in Western settings. Specifically, the positive association between BAP levels and TD sibling adjustment difficulties reported in Western cultures (e.g. Pisula and Ziegart-Sadowska 2015; Petalas et al. 2012) has never been explored in Chinese populations, nor has the utility of the diathesis-stress model been tested in this culture. Societal perceptions of disability, interpretation of Western-developed concepts of adjustment, and parents’ perceptions of their child’s behaviour all vary between Chinese and Western cultures (Phinney et al. 2000; Tsai 2016). As cultural factors shape family experience (Sage and Jegatheesan 2010; Lin et al. 2011; Tsai 2016) the utility of the diathesis-stress model might differ from one country to another. It is therefore important to explore the extent to which this model characterises sibling experience in Chinese as well as Western contexts in order to inform support practices in Chinese societies.

One of the challenges in Western sibling diathesis-stress studies to date is determining the role that parental BAP plays. Previous research has partially supported the role of parents’ BAP traits as a genetic vulnerability factor within a diathesis-stress model. For example, Orsmond and Seltzer (2009) reported an interaction between parents’ BAP traits and an environmental stressor (sibling life events) to predict sibling depressive symptoms, but other interactions that they explored (e.g. parents’ BAP and behaviour problems in the child with autism) did not predict sibling outcomes. However, parental BAP traits might also contribute to apparent poorer sibling outcomes by creating measurement issues. For example BAP traits in parents of children with ASD might affect sensitivity to maladaptive behaviour, in turn influencing their perceptions and reporting of their children’s adjustment (Petalas et al. 2012; Orsmond and Seltzer 2009). To date, however, the relations between parents’ BAP level and how they evaluate their children’s behaviour have only been explored in Western research (e.g. Petalas et al. 2012). It has previously been reported that Chinese parents adopt higher standards of expected child behaviour than Western parents (Chao 1994; Shek and Chan 1999; Porter et al. 2005). However, Lau et al. (2013) notes that there appears to be a consistency in the manifestation of autistic traits across Chinese and Western studies. Overall, it is not clear whether BAP levels will affect parents’ reports of their child’s behaviour differently in the two cultures.

This research seeks to fill existing gaps in our knowledge by investigating the role that genetic liability (BAP level) plays in sibling adjustment. Specifically, it explored how BAP level is associated with adjustment in TD siblings in the United Kingdom (UK) and Taiwan. It also explores the relations between parental BAP and sibling adjustment (employing both parental and self-report scores), in order to explore whether parental BAP appears to be related to sibling adjustment, or to the parent perceptions of sibling adjustment. The extent to which the diathesis-stress model provides a good explanation of factors associated with sibling adjustment in both Western and Chinese cultural settings was also investigated. Based on previous research, we predicted that TD siblings in both countries with higher level of BAP would show greater adjustment difficulties than those with lower levels of BAP. We also hypothesised that parents with higher BAP levels would report greater adjustment difficulty in their TD children than would parents with lower BAP levels, and aimed to explore whether this effect would differ across the two countries. Finally, we predicted that the diathesis-stress model would be moderately supported in the Western setting, with some significant interactions between BAP level and environmental stress predictive of sibling adjustment. The extent to which this model would provide a satisfactory framework in the Chinese setting was also explored.

## Methods

### Participants

The inclusion criteria for the study were (a) families with a child with ASD and another TD sibling between 7 and 18 years old living at home; (b) formal ASD diagnosis in the child with ASD, as indicated by parental report; (c) TD siblings and mothers had sufficient Chinese or English skills (as appropriate) to participate in the research. As the study was focused on the role of culture in sibling experiences, inclusion was restricted to families who self-identified as being of UK/Irish origin in the UK and of Chinese/Taiwanese origin in Taiwan. Whilst the resultant samples do not then fully represent the ethnic diversity which exists within the two research settings, such criteria allowed for examination of the role of culture in sibling adjustment. Families were excluded if (a) the child with ASD did not have a formal diagnosis or was waiting for a diagnosis; (b) the participating siblings had a suspected ASD diagnosis. Potential sibling participants were not formally screened for inclusion in this respect, but project materials made inclusion criteria clear, and siblings were excluded if mothers indicated that an ASD diagnosis was suspected.

Birth order and age have been found to relate to sibling adjustment (Stoneman 2005; Roeyers and Mycke 1995; Breslau 1982). For consistency, therefore, in families with more than one TD sibling, the one whose age was closest to that of the child with ASD was asked to participate.

There were 89 and 85 mother-TD sibling dyads of questionnaires received from Taiwan and the UK respectively. However, if participants had not completed more than 20% of the whole research survey, their data were excluded from the analyses. This applied to 10.1% participants in Taiwan and 11.8% in the UK. The final sample included in the analysis was therefore 80 and 75 mother-TD sibling dyads from Taiwan and the UK respectively. All the mothers were biological mothers. The Taiwanese sample were 98.8% Taiwanese and 1.2% Chinese, while 95.9% of participants in the UK sample were British and 4.1% were Irish.

The two cultural groups did not differ from each other in terms of the age and gender of the ASD and TD siblings, nor in the symptom severity of the children with ASD and the proportion of children falling into each diagnostic sub-group. Children with ASD in Taiwan had significantly higher rates of combined intellectual disability, while their UK counterparts had significantly higher rate of comorbid diagnoses. These findings may reflect differences which exist in clinical/diagnostic practice and how children’s behaviours are viewed between cultures (e.g. Norbury and Sparks 2013). Mothers in the UK also reporting significantly higher education levels, job positions and subjective wealth than the Taiwanese mothers (see Table 1).

**Table 1:** Parent and child characteristics of Taiwanese (TW) and the United Kingdom (UK) samples

		TW sample (n = 80)	UK sample (n = 75)	Statistic
Parental characteristics				
Relationship status	(% living with partner)	94.9%	82.4%	6.05*
Level of education	(% university or above)	35.4%	68.9%	19.1**
Employment status	(% full-time)	46.3%	23.0%	20.0***
Job description	(% professional/non-manual skilled)	44.9%	79.1%	21.9**
Subjective wealth	(% manage alright)	49.3%	72.2%	26.4***
Number of children	Mean (*SD*) [range]	2.3 (0.5) [2–4]	2.7 (0.8) [2–5]	−3.29**
Children’s characteristics				
ASD sib age in years	Mean (SD) [range]	12.2 (3.6) [4.0–21.5]	11.2 (3.5) [5.0-21.3]	1.65
TD sib age in years	Mean (*SD*) [range]	12.7 (2.8) [7.2–18.0]	12.7 (2.4) [8.6–17.7]	−0.05
ASD and TD sib age difference	(ASD-TD) [range]	−0.52 (3.3) [−8.1 to 10.7]	−1.6 (3.5) [−9.9 to 6.0]	1.82
ASD sib gender	(% male)	88.2%	87.8%	0.004
TD sib gender	(% male)	41%	37.3%	0.22
ASD sib diagnosis	(% ASD)	74.7%	60.8%	3.3
ASD severity *(SRS T scores)*	Mean (*SD*) [range]	78.8 (8.8) [55–90]	79.9 (9.2) [55–90]	−0.78
ASD sib presence of ID	(% yes)	47.5%	24%	22.7***
ASD sib comorbid other diagnosis	(% yes)	18.9%	45.9%	12.3***
ASD sib type of school attended	(% special education school)	16.5%	28.4%	30.5***
Study variables				
ASD sib severity *(SRS T scores)*	Mean (*SD*) [range]	78.8 (8.8) [55–90]	79.9 (9.2) [55–90]	−0.78
Parent AQ scores	Mean (*SD*) [range]	17.8 (7.5) [5–37]	12.9 (7.7) [2–42]	3.96***
TD sib AQ scores	Mean (*SD*) [range]	66.6 (21.5) [30–114]	52.84 (33.6) [4–132]	2.97**
Life events				
Negative events numbers	Mean (*SD*) [range]	3.7 (2.6) [0–9]	4.6 (2.8) [0–11]	−2.15*
Negative events impact	Mean (*SD*) [range]	7.8 (5.4) [1–22]	9.8 (6.6) [0–26]	−1.99*

*TD sib* typically developing sibling, *ASD sib* children with ASD, *ID* intellectual disability, *SRS* social responsiveness scale, *AQ* autism spectrum quotient

*p < .05; **p < .01; ***p < .001

### Procedure

Ethical approval was obtained from the authors’ institution prior to commencement of the study. There were three major recruitment routes. Firstly, parents were contacted via organizations/schools/support groups (with additional ethical approval obtained as required) and secondly the study was publicized online direct to potential participants. Due to differences in standard research practice and in participant expectations in Taiwan and the UK, there was a third recruitment route in Taiwan via hospital and psychiatry clinics. Permission from the Institutional Review Board (IRB) was obtained in each hospital, and potential participant families were then contacted via their clinician. When expressions of interest were received from parents, it was ascertained that the family met the recruitment criteria, and one of the researchers spoke with the parents or TD siblings to clarify the inclusion criteria/research procedure if necessary.

Participant families were supplied with parent and sibling project information and consent/assent sheets via post, email, social networking sites, or schools. Informed consent/assent was obtained from the parent and the TD sibling. Families were then sent two bound packs of questionnaires (one for the mother and the other for the TD sibling), each containing full instructions, and a stamped addressed envelope for the mother to return the completed questionnaires. TD siblings were provided a blank envelope to seal their questionnaires in and they then returned their sealed pack via their mother to ensure the confidentiality of these responses.

### Measurements

This was part of a wider study of sibling adjustment (Tsai 2016; Tsai et al. 2016), with findings from a subset of measures reported here. Based on the diathesis-stress model two measure of genetic liability (BAP characteristics in the sibling and the mother), two of environmental stress (number of negative life events experienced by the sibling; symptom severity in the child with ASD), and one of sibling adjustment were used. In addition, a parent questionnaire was used to collect a variety of demographic information (see Table 1).

#### Genetic Liability

The BAP of parents was measured using the AQ (Baron-Cohen et al. 2001) and the AQ-Chinese (Lau et al. 2013; Liu 2008). The AQ-Adult is a 50-item self-report inventory to assess the level of the autistic traits in the general population. This approach of quantifying autistic characteristics produces scores for ten items for each of five domains: social skills, attention switching, attention to detail, communication and imagination. Respondents are asked to rate the degree to which they believe they show the behaviour described in the item, with the responses ‘definitely agree’ or ‘slightly agree’ subsequently scored as ‘1’, and the responses ‘slightly disagree’ or ‘definitely disagree’ subsequently scored ‘0’. A high score [the suggested cut-off is a total score of 32 out of 50 for the AQ (Baron-Cohen et al. 2001) and 30 out of 50 for the AQ-Chinese (Liu 2008)] may be associated with a diagnosis of high-functioning ASD or Asperger syndrome. For the current sample, the internal consistency was 0.89 for the Taiwanese sample and 0.97 for the UK sample.

In the present research the AQ-Adult was used to explore mothers’ BAP and how it related to TD siblings’ adjustment outcome. Although the factor structure and scoring of the AQ Chinese (Lau et al. 2013) is somewhat different to that of the AQ, within the present study the 50 items of the original English version of the AQ-Adult and the dichotomous scoring method were used for both the Taiwanese and the UK parents, in order that the Taiwanese and UK data could be directly compared. Potential concerns about the cultural suitability of the AQ are addressed in the “Discussion” section.

BAP in the TD siblings was assessed via parent report using the 50-item Autism Spectrum Quotient Child/Adolescent (depending on the age of the TD sibling) version (AQ-Child/Adol) (Auyeung et al. 2008; Baron-Cohen et al. 2006) and the Chinese version (Lai 2009; Chan and Liu 2008). Both the AQ-Child and the AQ-Adol have the same five domains as the adult version. Again each domain is evaluated from responses to ten items, with a total of 50-items per questionnaire. The child and adolescent versions are directly comparable and also relate to the adult self-report AQ, showing similar scoring patterns (Auyeung et al. 2008; Baron-Cohen et al. 2006).

Due to its use of a 4-point Likert scale scoring, which is different to the AQ-Adult and the AQ-Adol, the cut-off score of AQ-Child is suggested to be 76 out of 150 (Auyeung et al. 2008). A cut-off of 30 is suggested for the AQ-Adol (Baron-Cohen et al. 2006). In the present research, a 4-point Likert scale was used with both the AQ-Child and the AQ-Adol, in accordance with the scoring used in other studies using AQ series measurements (e.g. Auyeung et al. 2008; Austin 2005; Hoekstra et al. 2007).

The Chinese version uses the same items, the same domains and the same scoring system as the English version. Despite the fact that the Chinese AQ-Child/Adol have been widely used in clinic settings, there are no published papers that provide clear information on its psychometric properties and cut-off scores. For the current study, the internal consistency of the AQ-Child was ⍺ = 0.93 and ⍺ = 0.97 in Taiwan and the UK respectively, and for the AQ-Adol it was ⍺ = 0.89 and ⍺ = 0.97 in Taiwan and the UK respectively.

#### Environmental Stressors

##### The Severity of Symptoms Shown by the Child with ASD

Autistic symptom severity in the child with ASD was assessed via mothers’ report using the 65-item Social Responsiveness Scale, 2nd Edition (SRS-2) (Constantino 2012) in the UK and the Chinese version of this (Gau et al. 2013) in Taiwan. Parents reported their child’s symptoms using a 4-point Likert scale to respond to statements which may or may not describe their child. The scale options range from ‘not true’ to ‘almost always true’ with higher scores indicating increased social dysfunction. Internal consistency for the present sample was ⍺ = 0.94 for both the Taiwanese and the UK samples. The suitability of the SRS-2 with Chinese populations has previously been demonstrated (Wang et al. 2012).

##### TD Sibling Life Experience

The presence of stressful life events was assessed by TD sibling self-report using the Child and Adolescent Survey of Experiences (CASE) (Allen et al. 2012) and a Chinese version translated by the present research team. The translation process followed the recommendations by Flaherty et al. (1988) and Guillemin et al. (1993).

The CASE relies on an individual’s interpretation of their life experiences over the previous 12 months. The TD siblings were given a list of events (38 items and 2 blank item to fill in themselves if they had any to add), and were asked to report whether they had experienced these events or not and then to rate the impact of the events using a 6-point scale from 1 (really good) to 6 (really bad). If they had not experienced the event listed, they circled the ‘no’ response and proceeded to the next life event. This approach allowed the respondents to decide whether the events were positive or negative to them; a total impact of positive and negative events in their life could then be produced. Hence, this response format recognizes that similar life experiences may be experienced as positive by some individuals and as negative by others. The individual’s perception and explanation of experiences has been suggested to have a crucial impact on the outcome (e.g. Jackson and Warren 2000). Hence, the present research incorporated this important implication of assessing TD siblings’ appraisal of their experience using total negative life events impact score rather than the cumulative number of life events.

The CASE has been shown to have satisfactory discriminability between community and clinical samples (Allen and Rapee 2009; Kercher et al. 2009) and associations with other interview-based measurements such as the Psychosocial Assessment of Child Experiences (Sandberg et al. 1993). The Kuder–Richardson-20 for the present UK and Taiwanese data on number of life events experienced was 0.66 and 0.67 respectively. The Cronbach’s alpha for the emotional impact of the experience was 0.93 in the UK and 0.96 in Taiwan.

#### Adjustment Outcome

TD sibling adjustment was assessed using both the parent report and the self-report version of the Strengths and Difficulties Questionnaire (SDQ) (Goodman et al. 1998) and the Chinese version (Liu et al. 2013). This is a 25-item measure covering emotional, conduct, hyperactivity/inattention, peer problems and prosocial behavior. By summing the scores of the first four subscales, users can create a ‘Total Difficulties’ score, where higher scores indicate greater difficulties, while higher ‘Prosocial Behavior’ scores reflect greater positive behavior. Although Taiwanese families completed SDQs containing all 25 items, Liu et al. (2013) has suggested a different factor structure than the original SDQ. Only the hyperactive subscale remains the same as the original self-report version SDQ, the composition of the remaining subscales is slightly different. In our analysis, the Taiwanese format was used for comparison with normative data to reflect the cultural factors emphasized in the present research. All other analyses were based on the original 25-item version of the SDQ to allow direct comparison between the Taiwan and the UK data.

Whilst the SDQ was originally designed for 11–16 years old, its use with younger children has been supported by other studies (Liu et al. 2013; Muris et al. 2003). In the present study the internal consistency in the parent-report SDQ for total difficulties scores was 0.60 in Taiwan and 0.88 in the UK, while in the TD sibling self-report it was 0.60 and 0.70 in Taiwan and the UK respectively.

### Analysis Plan

To maintain the fundamental social/cultural characteristics of each sample, we did not match groups on demographic variables, such as parental age and family size, as such variables can vary across cultures. For example, the average number of children per family is 2.65 in Taiwan and 1.7 in the UK (Office for National Statistics 2012; Directorate General of Budget Accounting and Statistics 2002).

Before the main analysis, all the data were checked for normality and homogeneity of variance. To explore similarities and differences in Taiwanese and the UK SDQ and BAP data, these were compared to normative data using t-tests. A series of correlation analyses were used to explore the role genetic liability plays in TD sibling adjustment. Bonferroni corrections for multiple comparisons were applied when more than 5 variables were examined (Curtin and Schulz 1998). Following the approach taken by Baron and Kenny (1986) for moderator analysis, hierarchical regressions were conducted to examine the extent to which genetic vulnerability (siblings’ and mothers’ BAP) moderated the relationship between environmental stressors (severity of symptoms in the child with ASD or impact of negative life events) and TD siblings’ adjustment outcome (SDQ total difficulties and prosocial behaviour). If the interaction effects were found to be significant, the moderator role of genetic vulnerability could be confirmed. The Taiwanese and the UK data were examined separately to examine the fit of the model in the two countries.

## Results

### Levels of TD Sibling Adjustment and Mother/TD Sibling BAP

The level of TD sibling adjustment and mother/TD sibling BAP was compared to explore similarities and differences between the countries and with normative data. The mean self-rated adjustment scores of TD siblings are reported in Table 2. With the exception of the conduct problem and hyperactivity/inattention subscales, the UK siblings evaluated themselves as having significantly more adjustment difficulties but also perceived themselves as having significantly higher prosocial behavior than their Taiwanese counterparts. In the UK sample all the mean ‘problems’ subscale scores, with the exception of conduct problems, were higher than the British normative data, with total difficulties [t(4301) = 7.99, p < .001, d = 0.93], emotional symptoms [t(4301) = 5.01, p < .001, d = 0.58], hyperactivity/inattention [t(4301) = 2.82, p < .01, d = 0.33], and peer problems [t(4301) = 14.48, p < .001, d = 1.69], indicating elevated adjustment difficulties. Prosocial behavior did not differ significantly from the British normative data. Siblings in Taiwan did not show significantly elevated adjustment difficulties compared to Taiwanese norms, and conduct problem scores were significantly lower than the norms [t(2750) = 4.31, p < .001, d = 0.49]. However, Taiwanese siblings did report significantly lower scores on the prosocial subscale than the norms [t(2750) = 9.02, p < .001, d = 1.02] (see Tsai et al. 2016 for further discussion of the SDQ data).

**Table 2:** Mean (SD) Sibling Strengths and Difficulties Questionnaire (SDQ) adjustment scores (self-rated)

	TW sample (n = 80)	UK sample (n = 75)	Between countries^a^	
			*t*-value	Cohen’s *d*
Total difficulties score	12.43 (5.27)	15.14 (5.26)	−3.19**	0.51
Emotional symptoms	2.97 (2.34)	4.03 (2.56)	−2.70**	0.43
Conduct problems	2.22(1.50)	2.71 (1.73)	−1.88	0.30
Hyperactivity/inattention	4.36 (2.11)	4.52 (1.81)	−0.53	0.08
Peer problems	2.65 (1.64)	3.88 (1.92)	−4.29***	0.69
Prosocial behaviour^b^	6.81 (2.21)	7.64 (1.70)	−2.62*	0.42

*p < .05; **p < .01; ***p < .001

^a^The same factor structure and number of items were used for both the Taiwanese and the UK SDQ data, to allow between-country comparison

^b^Higher scores indicate more prosocial behaviour

Comparison of the mothers’ BAP levels between the two countries revealed a significant difference (see Table 1), with Taiwanese mothers reporting significantly higher BAP levels than the UK mothers [t(149) = 3.96, p < .001, d = 0.64]. Taiwanese siblings’ BAP level was significantly higher than that of the UK siblings [t(120) = 2.97, p < .01, d = 0.48]. Further comparison with country-specific cut-off scores (32 for the UK and 30 for the Taiwanese) showed that 4.1% of UK mother and 6.5% of Taiwanese mother self-rated above the cut-off. As for the TD siblings, 23.6% of UK siblings were rated above the cut-off score of 76 for AQ-Child and 30 for the AQ-Adol. A cut-off score was not available for comparison with the Taiwanese sibling AQ data.

### Relation Between BAP Levels (TD Sibling and Mother) and Sibling Adjustment

A series of correlations were used to test the hypotheses that siblings with higher level of BAP (as reported by mothers) would show greater adjustment difficulties, and that parents with higher BAP levels would report greater adjustment difficulties in their TD children than would parents with lower BAP levels. The relations between mothers’ BAP levels, TD sibling BAP levels (as reported by mothers), and TD sibling SDQ scores (as reported by mothers and siblings) in the two countries are reported in Table 3.

**Table 3:** Intercorrelations among BAP level and SDQ

	Mother-rated SDQ		TD sibling self-report SDQ	
	Total difficulties	Prosocial behavior	Total difficulties	Prosocial behavior
Taiwan				
Mother BAP	0.30*	−0.23	0.04	−0.06
TD sibling BAP	0.61*	−4.9*	−0.05	−0.05
UK				
Mother BAP	0.11	−0.02	0.12	−0.06
TD sibling BAP	0.35*	−0.20	0.30	−0.11

*BAP* broader autism phenotype, *SDQ* strengths and difficulties questionnaire

*After Bonferroni correction new p value = 0.01

Significant correlations were found between mother-rated SDQ and BAP level. Mother-rated SDQ total difficulties scores were significantly positively correlated with mothers’ BAP level in the Taiwanese but not in the UK data, indicating that Taiwanese mothers who had higher self-reported BAP level also tended to rate their TD children as displaying more difficulties. Significant correlations between mother-rated SDQ and TD sibling BAP levels revealed that mothers’ view of elevated adjustment difficulties on the SDQ was significantly associated with higher BAP levels in TD siblings in both countries. Moreover, mother-rated SDQ prosocial behaviour was significantly negatively associated with TD siblings’ BAP levels in Taiwan but not the UK.

There was no significant correlation found between sibling self-report SDQ scores and BAP levels (either the mothers’ or the TD siblings’) in either the Taiwanese or the UK data.

### Moderator Analysis

Regressions were conducted to evaluate the hypothesis that TD siblings’ BAP level would moderate the relations between environmental stressors and adjustment outcome, at least in Western settings. All the data, except the dependent variable, were centred, to reduce multicollinearity between the variables. There were no violations of regression assumptions (including multicollinearity). Demographic variables (as listed in Table 1) were checked for significant correlations with outcome variables in each country. Only the age difference between ASD and TD siblings in Taiwan was found to significantly relate to TD siblings’ total difficulties scores [*r*(71) = .23, p = .049], but none of the demographic variables were significantly associated with TD siblings’ adjustment outcome in the UK. Hence, sibling age difference was included in the first step of the regression models for Taiwanese data reported below. With this exception, all variables entered into the models were identical for the two countries.

Separate subscales of the SDQ were utilised as outcome measures within the subsequent regression models. Firstly, the severity of ASD was examined as an environmental stressor and was entered at Step 1. The diathesis variables (siblings’ and mothers’ BAP level) were entered at Step 2. The interaction variables were entered at Step 3 (sibling’s BAP score × severity of ASD; mothers’ BAP score × severity of ASD) to test the diathesis-stress model prediction. These regression analyses are presented in Tables 4 and 5.

**Table 4:** Regression models predicting sibling self-report SDQ with severity of ASD as stressor in Taiwan

Predictors	Total difficulties	Emotional Symptom	Conduct problem	Hyperactivity/inattention	Peer problem	Prosocial behavior
Step 1						
Age difference between siblings	0.225	0.184	0.047	0.228	0.183	−0.199
Severity of ASD	−0.141	−0.152	−0.116	−0.097	−0.057	0.120
△*R* ^2^	0.059	0.047	0.014	0.053	0.033	0.045
△*F*	2.097	1.639	0.466	1.889	1.142	1.588
Step 2						
Age difference between siblings	0.233	0.182	0.083	0.228	0.181	−0.199
Severity of ASD	−0.146	−0.164	−0.117	−0.101	−0.050	0.124
TD sib BAP	−0.094	−0.068	−0.253	−0.026	0.062	0.032
Mother BAP	0.091	0.149	0.106	0.053	−0.091	−0.056
△*R* ^2^	0.010	0.019	0.053	0.002	0.008	0.003
△*F*	0.357	0.645	1.832	0.081	0.255	0.092
Step 3						
Age difference between siblings	0.233	0.187	0.061	0.238	0.181	−0.168
Severity of ASD	−0.149	−0.174	−0.094	−0.113	−0.053	0.094
TD sib BAP	−0.007	0.040	−0.161	−0.043	0.119	−0.150
Mother BAP	0.087	0.157	0.040	0.083	−0.092	0.041
Severity of ASD × TD sib BAP	−0.179	−0.225	−0.180	0.029	−0.119	0.359*
Severity of ASD × mother BAP	−0.032	−0.087	0.175	−0.093	−0.025	−0.228
△*R* ^2^	0.032	0.066	0.022	0.006	0.014	0.069
△*F*	1.104	2.375	0.778	0.195	0.483	2.474
Total *R* ^2^	0.101	0.131	0.089	0.062	0.055	0.117
F(6,63)	1.175	1.579	1.024	0.688	0.611	1.395

*TD sib* typically developing sibling, *ASD sib* children with ASD, *BAP* broader autism phenotype, *SDQ* strengths and difficulties questionnaire

*p < .05

**Table 5:** Regression models predicting sibling self-report SDQ with severity of ASD as stressor in the UK

Predictors	Total difficulties	Emotional symptom	Conduct problem	Hyperactivity/inattention	Peer problem	Prosocial behavior
Step 1 environmental stressors						
Severity of ASD	−0.099	−0.031	−0.213	−0.085	0.041	0.227
△*R* ^2^	0.010	0.001	0.045	0.007	0.002	0.051
△*F*	0.708	0.070	3.381	0.513	0.119	3.853
Step 2 genetic liability						
Severity of ASD	−0.165	−0.114	−0.221	−0.099	−0.008	0.262*
TD sib BAP	0.323**	0.311*	0.081	0.112	0.297*	−0.162
Mother BAP	0.038	−0.155	0.090	0.093	0.142	0.003
△*R* ^2^	0.106	0.096	0.017	0.025	0.121	0.025
△*F*	4.136*	3.689*	0.633	0.876	4.760*	0.927
Step 3 interaction variables						
Severity of ASD	−0.265*	−0.190	−0.272*	−0.138	−0.101	0.221
TD sib BAP	0.470**	0.404*	0.161	0.164	0.457**	−0.109
Mother BAP	−0.020	−0.208	0.063	0.067	0.100	−0.025
Severity of ASD × TD sib BAP	−0.277*	−0.167	−0.153	−0.096	−0.312*	−0.096
Severity of ASD × mother BAP	0.184	0.211	0.070	0.095	0.072	0.109
△*R* ^2^	0.070	0.050	0.018	0.012	0.069	0.014
△*F*	2.883	1.955	0.658	0.420	2.856	0.523
Total *R* ^2^	0.186	0.147	0.081	0.044	0.192	0.090
F(5,67)	3.060*	2.31	1.177	0.613	3.177*	1.333

*TD sib* typically developing sibling, *ASD sib* children with ASD, *BAP* broader autism phenotype, *SDQ* strengths and difficulties questionnaire

*p < .05; **p < .01

Twelve separate regression models were initially examined, six in each of the two countries. The procedure above was followed for each of the models, with one of the five SDQ subscales used as outcome measure in each model, and SDQ Total Difficulties score also used as an outcome measure.

**Fig. 1:**
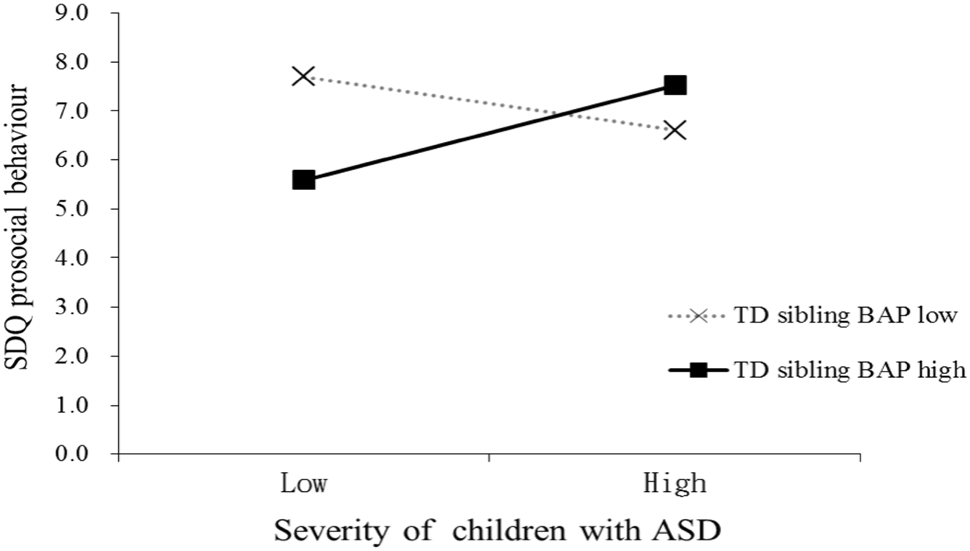
Interaction between the severity of symptoms of children with ASD and the TD siblings’ BAP level in predicting TD siblings’ self-report prosocial behaviour in Taiwan. *Note* low = 1 SD below the mean; high = 1 SD above the mean

One of the models using data from Taiwan (Table 4) was significant.^1^ In relation to TD siblings’ prosocial behaviour, a significant interaction between sibling BAP level and symptom severity in the child with ASD was evident: for TD siblings with lower levels of BAP (1 *SD* below the mean), their prosocial behaviour on the SDQ was negatively related to the severity of symptoms in the child with ASD. That is, TD siblings of less severely affected children with ASD displayed more prosocial behaviour than siblings of more severely affected children with ASD. However, this pattern was reversed in TD siblings with higher levels of BAP (1 *SD* above the mean) (Fig. 1). None of the other Taiwanese regression models were significant.

**Fig. 2:**
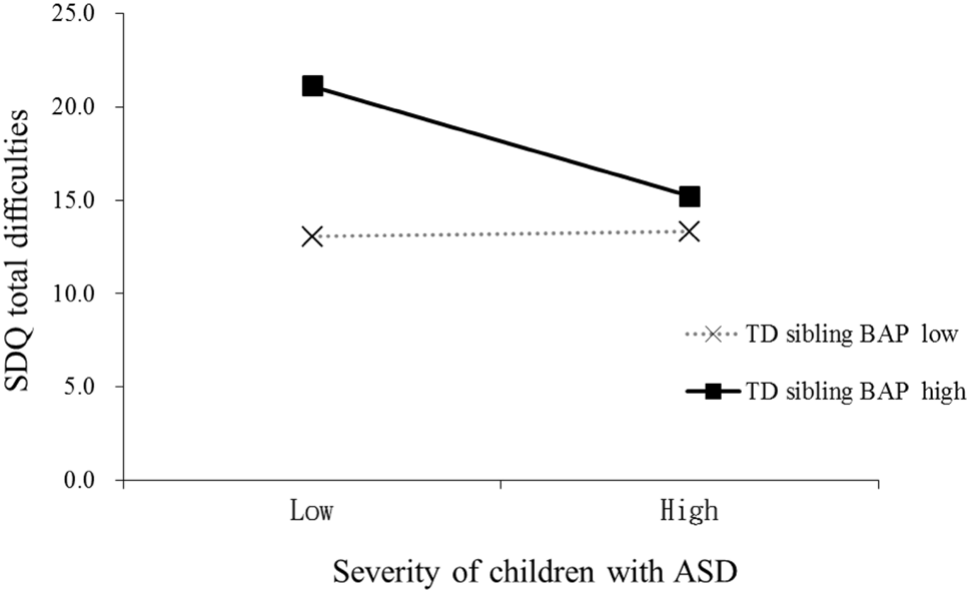
Interaction between the severity of symptoms of children with ASD and the TD siblings’ BAP level in predicting TD siblings’ self-report total difficulties in the UK. *Note* low = 1 SD below the mean; high = 1 SD above the mean

**Fig. 3:**
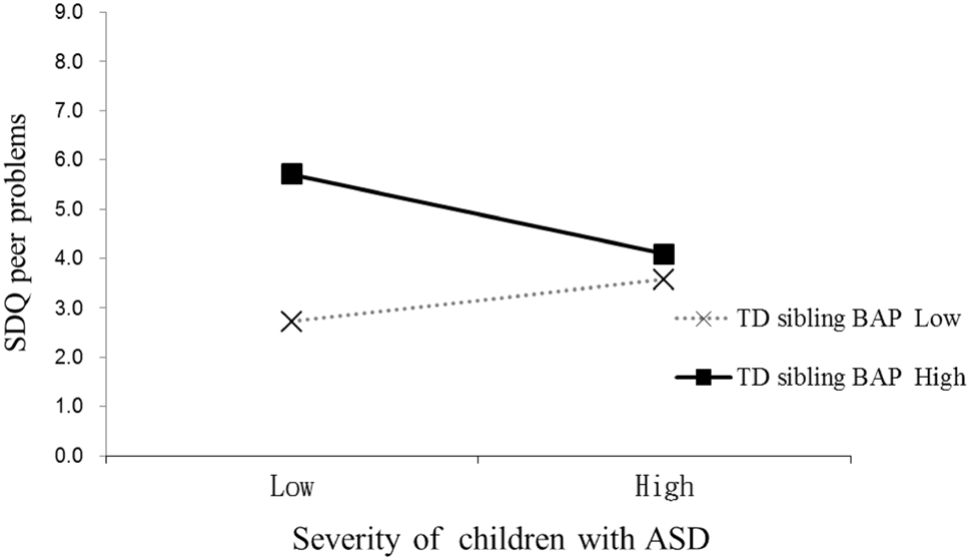
Interaction between the severity of symptoms of children with ASD and the TD siblings’ BAP level in predicting TD siblings’ self-report peer problem in the UK. *Note* low = 1 SD below the mean; high = 1 SD above the mean

In the UK sample, two significant interactions were found in the regression models (Table 5). The TD sibling BAP level moderated the influence of the severity of symptoms in the child with ASD on TD siblings’ total difficulties and sibling peer problems: for TD siblings with lower levels of BAP, their total difficulties and peer problems rating on the SDQ were positively related to the severity of symptoms in the child with ASD. That is, TD siblings of less severely affected children with ASD displayed fewer total difficulties and peer problems than siblings of more severely affected children with ASD. However, this pattern was reversed in TD siblings with higher levels of BAP (Figs. 2, 3). The other UK regression models were not significant.

An identical procedure was then used in the regression analyses to explore the interaction between sibling/mother BAP level and negative life events impact, none of the interactions were statistically significant either in the Taiwanese or the UK model. Hence, this analysis is not reported on further here.

## Discussion

The present research adds to the few cross-cultural comparisons between Chinese and Western families of children with autism to date, looking at TD siblings’ adjustment and the influence of BAP in relation to adjustment. With only a limited number of significant interaction effects found between TD siblings’ BAP level and severity of child with ASD, and an unexpected pattern in these interaction effects, the use of a diathesis-stress model as a research framework was only partially supported in both cultural settings. We also found that different adjustment outcomes were associated with BAP traits in the two countries. The significant links between mothers’ ratings of TD sibling adjustment and BAP (their own and the TD siblings’) provide both insight into the extent to which genetic liability might influence sibling adjustment, and raise questions about the role of different family members’ perspectives on sibling adjustment. This is an important finding for interpreting study designs which rely on parental report measures.

Comparison with norm data indicated that Taiwanese siblings were fairly well adjusted according to their self-report, whereas UK siblings reported elevated difficulties on all scales with the exception of conduct problems and prosocial behaviour compared to normative data. A previous UK study similarly found that the proportion of TD siblings of children with autism that fell within the clinical range on the peer problems scale was significantly higher than in the normative data (Hastings and Petalas 2014), although overall they reported fewer significant differences from the normative data than was found in the present study. There is little existing literature on Chinese siblings of children with autism, but our data contrast with the work of Lin (2012) who reported depression scores higher than the clinical cut-off in half of TD siblings surveyed (total sample = 29). An explanation for this might lie in the fact that, compared to our study, Lin’s included participants of a wider, and older age range (11–27 years), and age has previously been found to relate to adjustment levels (see Stoneman 2005 for discussion).

What is the meaning of the different levels of self-reported adjustment between our UK and Taiwan samples? There are three possible interpretations. One is that Taiwanese siblings do in fact show better adjustment than their UK counterparts, with findings genuinely representing a culturally-driven difference in adjustment in the two countries. The second possibility is that it reflects differences between the two groups which are not directly related to culture. Since there were more children with ASD with a co-morbid diagnosis in the UK, it is possible that the UK siblings faced more challenging situations than their Taiwanese counterparts (though this might also simply reflect cultural differences in diagnostic practices). A final possibility is that the findings do not necessarily reflect a difference in the actual level of sibling adjustment, but may instead result from culturally-specific pressures which impact on responses to questionnaires. Lalwani et al. (2006) concluded that people in collectivist countries are more likely to display socially desirable responding in order to present oneself in a culturally accepted and approved light than those from individualistic countries. Such a tendency might be exacerbated in families of children with ASD, where efforts to gain societal acceptance might be greater.

In the light of this final interpretation, the lower than average siblings’ prosocial behaviour scores in the Taiwanese sample are particularly noteworthy. They appear to contradict Chinese cultural norms, which emphasise and value behaviour involving positive social interactions and fulfilling the expected social roles (Oyserman et al. 2002). Our finding may reflect the effect of the still-prevalent social stigma of having a child with a disability in Chinese culture (e.g. Huang et al. 2009) and the family dilemmas involved in seeking social support (Chang and McConkey 2008), perhaps making it more difficult for Taiwanese siblings to socialise and develop friendships.

The significant relations between TD sibling BAP scores and mother-rated sibling adjustment on the SDQ supports previous findings (e.g. Petalas et al. 2012; Meyer et al. 2011; Mohammadi and Zarafshan 2014), indicating that higher levels of BAP traits are associated with adjustment difficulties. However, although this partially supported our hypothesis, our findings further suggest that the strength of this relation depends on the informants involved. There were no significant associations found between siblings’ BAP and their adjustment when we looked at siblings’ own ratings on the SDQ. Studies to date which have reported a significant direct association between sibling BAP and adjustment (e.g. Petalas et al. 2012; Meyer et al. 2011; Mohammadi and Zarafshan 2014) have only used a single informant (parent). The significant associations between mother-rated measures in contrast to a lack of association with a sibling-rated measure, might reflect some uni-rater response bias, or simply that greater years of experience that make mothers more accurate judges of their child’s behaviour. Alternatively, it may reflect the fact that siblings are better able to report on a wider range of behaviours and internal emotional aspects, whereas parents are limited to more directly observable behaviours and have to use the same evidence to rate both scales (AQ and SDQ).

We hypothesised that mothers with higher BAP levels would report higher sibling adjustment difficulties. In fact, the significant positive relation found between mothers’ BAP and mother-rated sibling SDQ adjustment difficulties in Taiwan but not in the UK, is a novel finding. This finding may have arisen because the higher BAP level in Taiwanese mothers results in higher levels of stress, (see e.g. Orsmond and Seltzer 2009; Petalas et al. 2012; Walton and Ingersoll 2015) which then impacts directly on TD siblings’ behaviour or the way in which mothers evaluate it. Our findings extend the previous research to ethnic Chinese families. Including maternal mental health measures would help in the future to further explore the pathways between maternal BAP and perceptions of sibling adjustment. Whilst the significant relation between the BAP and the SDQ might suggest some overlap of concepts in the two measures (e.g. in relation to peer interaction), it is noteworthy that significant associations between the two measures were not always found, and that the assumptions of multicollinearity were not found to be violated.

While the diathesis-stress model has previously been used in Western settings (e.g. Meyer et al. 2011; Mohammadi and Zarafshan 2014; Orsmond and Seltzer 2009; Petalas et al. 2012; Walton and Ingersoll 2015), our research has applied it for the first time in a Chinese cultural context. We predicted that it would be moderately supported, at least in the Western setting. In fact, the utility of the model in explaining TD siblings’ adjustment outcome was not robustly supported in the present study, with only 3 out of a possible 24 models found to be significant, and direction of effects opposite to that expected. In terms of number of significant interaction effects, this was a similar level of support to that found by both Orsmond and Seltzer (2009) and Petalas et al. (2012), with 3 out of 12 and 2 out of 20 interactions tested found to be significant, respectively. Although the cumulative findings for these studies do suggest that this is a model worthy of continued exploration, as Petalas et al., note, with the number of models tested, the possibility of Type I errors remains. In common with the sibling research more broadly, consistency in assessment measures across studies, larger sample sizes, and increased sample diversity would allow a more extensive exploration of the diathesis-stress model in the future. Additionally, a measure of challenging behaviour in the child with autism, rather than a measure of symptom severity (as used in the present study) would be useful in future studies to explore whether and how this interacts with genetic liability to impact the sibling in Chinese as well as Western contexts.

In relation to the specific nature of the interaction effects found to be significant, a number of issues merit further discussion. Firstly, while the interaction of sibling BAP with environmental stress (severity of symptoms in the child with ASD) correlated with the siblings’ behaviour problems (total difficulties and peer problems) in the UK, it was siblings’ prosocial behaviour that was affected in Taiwan. Although these were different significant interaction terms found in the two countries, together they indicate that the BAP had stronger associations with TD siblings’ social domains (e.g. prosocial behaviour and peer problems) than other behavioural domains (e.g. hyperactivity). This is consistent with research suggesting that BAP related traits tend to make TD siblings more vulnerable to very mild to significant difficulties in emotional understanding compared to siblings of children without ASD (Cassel et al. 2007; Yirmiya et al. 2006; Meadan et al. 2010).

A second issue is that, while our findings were supportive of the general notion that the interactions between genetic vulnerability and environmental stress were associated with TD sibling outcome in both cultural settings, the direction of these interaction effects was in fact the opposite of that predicted by the diathesis-stress model. Previous studies have consistently reported the positive relations between elevated adjustment difficulties and TD siblings BAP level when in the presence of the high stressors (e.g. Meyer et al. 2011; Petalas et al. 2012; Orsmond and Seltzer 2009; Walton and Ingersoll 2015). For example, Petalas et al. (2012) reported that the TD siblings with higher BAP level and ASD siblings who displayed more behaviour difficulties showed higher risk of adjustment difficulties than siblings with lower BAP level and siblings with fewer behaviour difficulties. Our findings indicated that for TD siblings with lower BAP, their adjustment difficulties increased, albeit only slightly, when there was a more severely affected child with ASD in the family. However, for siblings with higher levels of BAP, the presence of an environmental stressor (a more severely affected child with ASD), was in fact associated with a reduction in adjustment difficulties. The reason for the difference in direction of interaction effects across studies is unclear.

Our own results might be explained by the finding that BAP is associated with sub-clinical difficulties in social, cognitive and emotional domains (Ben-Yizhak et al. 2011; Gamliel et al. 2009; Pisula and Ziegart-Sadowska 2015), and so it may have been the case that siblings with lower levels of BAP made more attempts than those with higher BAP levels to socialise with the child with autism, and were aware of, and hence affected by, any increase in stressors around them. It might also have been the case that for TD siblings with higher levels BAP traits, the presence of an environmental stressor (a more severely affected child with ASD), was associated with a reduction in adjustment difficulties, because those particular TD siblings benefitted from autism-intervention strategies and parent support intended for their affected sibling (Walton and Ingersoll 2015). However, to distinguish between these possible explanations further studies are still required.

The present research did not find that sibling negative life events had a significant moderating role, although this was previously found by Orsmond and Seltzer (2009). Again, there were differences in measurement (e.g. sibling adjustment outcome measures), and Orsmond and Seltzer included some life events which could have been perceived as positive by the siblings (e.g. parent beginning a new job), whereas our own analysis only included life events which the siblings perceived of as negative. As before, these differences don’t clearly explain the difference in findings across the two studies. Nevertheless, in our own data, life events in conjunction with other variables did predict TD siblings’ adjustment in two countries (see Tsai et al. 2016).

The above proposed explanations for our findings await verification in future studies. The present research, nevertheless, leads to tentative suggestions for how caregivers or clinicians might best support siblings in the respective populations. In both countries, the impact of TD siblings’ BAP level on their maternal-reported adjustment is evident, suggesting that siblings with high BAP levels may need particular support, although as the diathesis-stress findings make clear, it is also important to consider the bigger picture, in terms of environmental stress. The results also highlight the importance of clinicians consider the influence of maternal BAP level when using mothers’ report of sibling adjustment, and collecting additional information from the sibling themselves wherever possible. UK siblings may also benefit particularly from support for peer and emotional problems.

### Limitations

As with other sibling research, this research faced challenges related to the use of parents as the main BAP information provider (Meyer et al. 2011; Ingersoll and Hambrick 2011; Orsmond and Seltzer 2009). In order to increase the validity of BAP assessment, using other informants, such as fathers or a teacher, might be useful (e.g. Möricke et al. 2016). Whilst a TD sibling self-report on their BAP traits would have allowed exploration of the consistency and diversity between parents’ and children’s points of view, none of measures identified were specifically designed for children/adolescents’ self-report, and there are ethical and sensitivity concerns around the gathering of such data. However, in using sibling SDQ data we did avoid issues around uni-rater response bias across all measures.

Given the sensitive nature of family research, possible volunteer bias is a concern. Families who participated in this research may have done so because they had particularly low or high levels of worry about their TD child’s adjustment. It is also important to note that there were slightly different approaches to recruiting participants in Taiwan and the UK, and again, although unavoidable due to the limited support systems and organisations in Taiwan, this may have had some impact on findings.

Whilst the two datasets were broadly similar in terms of demographics, there were some key differences. For example, UK mothers did show potentially higher social economic status than their Taiwanese counterparts. Whilst the decision not to match groups did mean that culture-specific demographic profiles were not lost, it did make it more difficult to disentangle the effect of culture versus demographics in explaining UK-Taiwanese differences in sibling adjustment. Indeed, often these cannot be disentangled, with culture, socio-economic status and family demographics all intertwined.

The mechanisms behind the BAP associations with siblings’ adjustment, and how this plays out in the daily lives of families cannot be fully explored through closed-question questionnaires alone, and should be used in tandem with methods such as interview to more fully understand the sibling experience (Tsai 2016). Furthermore, siblings’ experiences and relationships with their siblings with autism are not static, but change with life stages (Orsmond and Seltzer 2007). It will be highly valuable if future diathesis-stress research could follow up TD siblings through different stages of their life.

Choosing culturally sensitive measures was important in this cross-cultural study, especially as previous research has suggested that when some of the measures from the present study were used in Chinese populations there was some support for a similarity of some concepts, but also some differences, related to different philosophical underpinnings (e.g. Liu et al. 2013; Lau et al. 2013). Future research should continue to use and develop culturally sensitive measurements, as it is clear from the present study that understanding cultural differences in perceptions of adjustment is vital if we are to support siblings effectively.

## Conclusions

Overall, the present research partially supported the continued use of the diathesis-stress model for framing and understanding the experiences of siblings of children with ASD in both Taiwan and UK contexts. The results generally confirmed the importance of considering the influence of TD siblings’ BAP level in predicting their adjustment outcome. Despite its limitations, this research has provided a picture of how variables operate in culturally-similar and culturally-specific ways in relation to TD siblings’ adjustment, as well as the influence of BAP in in both Chinese and Western cultural settings. Future research that evaluates TD siblings’ adjustment, using a variety of informants, including self-report from the siblings is also highly recommended.

## Footnotes

1 None of the Taiwanese models were significant when the Chinese-specific factor structure of the SDQ was used as the outcome variable.
